# Rapid phenotypic individualization of bacterial sister cells

**DOI:** 10.1038/s41598-017-08660-0

**Published:** 2017-08-16

**Authors:** Sander K. Govers, Antoine Adam, Hendrik Blockeel, Abram Aertsen

**Affiliations:** 1 KU Leuven, Department of Microbial and Molecular Systems (M²S), Faculty of Bioscience Engineering, 3001 Leuven, Belgium; 2 KU Leuven, Department of Computer Science, 3001 Leuven, Belgium; 3 Microbial Sciences Institute, Yale University, West Haven, CT USA

## Abstract

A growing bacterium typically divides into two genetically identical and morphologically similar sister cells and eventually gives rise to a clonal population. Nevertheless, significant phenotypic differentiation among isogenic cells frequently occurs, with the resulting heterogeneity in cellular behavior often ensuring population level growth and survival in complex and unpredictable environments. Although several mechanisms underlying the generation of phenotypic heterogeneity have been elucidated, the speed with which identical sister cells tend to phenotypically diverge from each other has so far remained unaddressed. Using *Escherichia coli* as a model organism, we therefore examined the timing and dynamics of phenotypic individualization among sister cells by scrutinizing and modeling microscopically tracked clonally growing populations before and after a semi-lethal heat challenge. This analysis revealed that both survival probability and post-stress physiology of sister cells shift from highly similar to uncorrelated within the first decile of their cell cycles. This nearly-immediate post-fission randomization of sister cell fates highlights the potential of stochastic fluctuations during clonal growth to rapidly generate phenotypically independent individuals.

## Introduction

Microbial proliferation is characterized by the generation of isogenic clones. Although cells in such clonal populations thus have the same range of genes at their disposal, they often display extensive phenotypic heterogeneity, defined as variability of a given trait or behavior in an isogenic population in a homogeneous environment^1^. In recent years, it has become increasingly more clear that this heterogeneity is not just a mere byproduct of stochastic or deterministic fluctuations in the molecular composition of individual cells^2, 3^, but instead often serves a more functional purpose^4^. As such, the generation of phenotypic heterogeneity has been implicated in increasing population-level fitness and functionality by permitting bet-hedging and/or division of labor strategies^5, 6^.

The molecular cues that can serve as initiators of phenotypic differentiation are known to range from stochastic fluctuations in cellular composition to the more deterministic uneven distribution of cellular features such as cell pole age (in rod-shaped bacteria)^4, 5^, and can be propagated by genetic feedback loops to establish transiently stable and inheritable phenotypic states^7^. Notwithstanding these insights, the potential rate of cellular differentiation remains largely unaddressed or is inspired by a small number of cases focusing on clearly defined low frequency switches (typically retained over at least a number of generations) between well-characterized phenotypic states^8–11^.

In this study, we therefore scrutinized the individualization dynamics between morphologically and genetically identical sister cells of the *Escherichia coli* model bacterium with respect to a more comprehensive and complex phenotype such as post-stress survival fate that has the potential of revealing even subtle stochastic intercellular differences.

## Results

### Stochastic survival-assay reveals randomized coupling of sister cell survival fates

**Figure 1:**
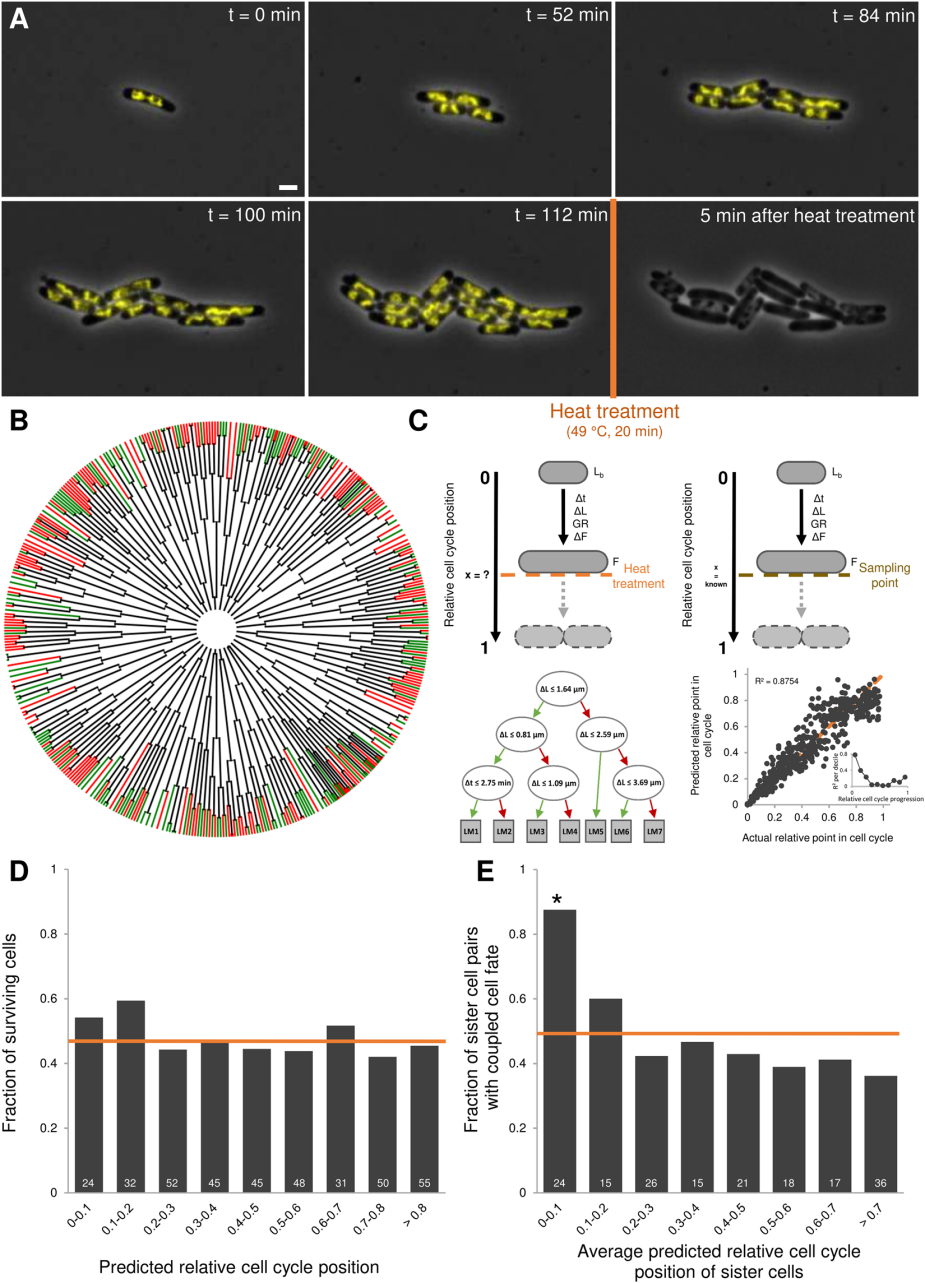
Single-cell level survival-assay reveals rapid sister cell individualization. (**A**) Representative images of a TLFM microscopy image sequence of growing MG1655 *hupA-yfp* cells at indicated times before and after heat treatment (49 °C, 20 min). Phase contrast images are superimposed with YFP epifluorescence images (reporting nucleoid dynamics). The scale bar corresponds to 2 μm. (**B**) Schematic representation of all observed microcolonies (n = 29) and cells (n = 821). Every end point in the tree represents one cell exposed to the heat treatment (n = 425); green tips: surviving cells, red tips: non-surviving cells. (**C**) Schematic representation of our survival-assay (top left) and sampling approach (top right; based on training data from unstressed cells). As cells grow, many measurable cellular attributes (Lb = length at birth, Δt = time since birth, ΔL = length increase since birth, GR = growth rate, ΔF = increase in cellular DNA content, F = cellular DNA content) can be employed to predict a cell’s relative position in its cell cycle at the moment of heat treatment (x). The model itself consists of 7 linear models (LM) preceded by a regression tree. Green arrows indicate a positive answer, red arrows a negative answer. The performance of the model was assessed by 10-fold internal cross-validation (n = 635; R² = 0.8754, p-value = 6.17 × 10^−221^, RMSE = 0.100). The bisector is shown as a dashed orange line. Inset displays the evolution of the R² value, calculated by examining the correlation between predicted and actual relative cell cycle progression per independent decile. (**D**) The fraction of cells surviving the heat treatment (49 °C, 20 min) binned by their predicted relative cell cycle position (n = 425). None of the individual bins differed significantly from the average of all cells (orange line, 45.4%, 95% confidence interval (CI) = [40.7%, 50.2%]; Fisher’s exact test, α = 0.01). Indicated in white is the total number of cells that was observed for each bin. (**E**) Fraction of sister cells with coupled cell fate (i.e. both cells either survive or die) is binned by the average predicted relative cell cycle position of the siblings (n = 172 sibling pairs). Indicated in white is the total number of sibling pairs that was observed for each bin. Orange line indicates the amount of coupling that would be expected by chance (50.4%, given the overall level of survival), asterisks indicate significant differences (Fisher’s exact test, α = 0.01).

To examine the temporal dynamics of cellular individualization and its potential phenotypic implications, we monitored growing *Escherichia coli* MG1655 *hupA-yfp* cells at the single-cell level by time-lapse fluorescence microscopy (TLFM) before and after the application of a heat treatment leading to the inactivation of approximately half of the cells. The chromosomally expressed HupA-YFP fusion protein serves as a nucleoid reporter that allowed us to keep track of chromosome replication and segregation during growth and division of the monitored cells prior to the heat treatment^12^, and evaluate whether any of these processes significantly affected survival and/or individualization. Before the heat challenge, single cells were monitored by TLFM during growth for approximately 4 generations into microcolonies consisting of 8–23 cells (Fig. 1A). These microcolonies were subsequently subjected to a heat treatment (49 °C for 20 min) and further monitored by TLFM for an additional 6 hours, allowing cellular survival, in our setup defined as cells being able to resume growth and subsequent division, to be determined (Fig. 1A). In total, the growth of 29 microcolonies was monitored before and after heat shock, registering 425 heat-shocked cells of which 45.4% were able to survive the heat treatment (Fig. 1B).

Since heat stress simultaneously targets multiple cellular processes^13^, the impact of potential epigenetic inheritance and/or predisposition effects on cellular survival odds (of ca. 50%) is likely to be negligible. Furthermore, an elaborate analysis of our survival-assay could not reveal the predominance of such effects on cell fate (Supplemental text and Fig. S1–2), further allowing survival to be considered as a stochastic cellular trait determined by stochastic variability between cells. In line with our expectations, even sister cells did not appear to have a tendency to share the same fate in our assay (49.7% as measured versus 50.4% as expected by chance given the overall frequency of survival, p-value = 0.79), indicating that each individual cell appeared to act as an independent entity, each with an equal chance to survive.

### Predictive cell cycle progression model reveals rapid individualization of sister cells

Given that sister cells (i.e. cells that share the same pedigree and have only recently originated from a common mother cell) displayed independent phenotypic behavior when challenged in our survival-assay, we set forward to further probe the underlying dynamics of this individualization process. To this end, we constructed and validated a predictive cell cycle progression model, based on unstressed training data of growing MG1655 *hupA-yfp* cells (Fig. 1C), to more carefully assess the timing of this differentiation with regard to their relative cell cycle progression. Construction of the model was required since (relative) cell cycle progression, due to its inherent stochastic nature and dependence on previous generations, cannot be accurately estimated by taking only a single parameter (e.g. time since birth) into account. The model allowed the quantification of relative cell cycle progression at the moment of heat shock as a number between 0 and 1 (which correspond to a cell’s birth and division, respectively), based on the time-resolved measurements of a number of cellular features (length at birth, time since birth, length increase since birth, growth rate, increase in cellular DNA content, total cellular DNA content; Fig. 1C). Although we initially reasoned that the visualization of chromosome replication and segregation would improve the performance of this model, other measurable factors, such as length increase and time since birth, were found to be most important in predicting cell cycle progression (Fig. 1C and Fig. S3). Overall, the model’s predictions proved relatively accurate (R² = 0.8754, p-value = 6.17 × 10^−221^, root mean squared error (RMSE) = 0.100), although its performance decreased in later cell cycle stages (Fig. 1C, inset).

We subsequently employed this model to more closely examine the uncoupling of sister cell fates as they progress through their corresponding cell cycles. In this analysis, sister cell fates are considered coupled when both cells share the same fate (i.e. both cells either survive or die) and uncoupled when sister cell fate differs (i.e. one cell survives and the other dies). Interestingly, whereas cell cycle progression itself had no influence on the fate of individual cells (Fig. 1D), freshly formed sister cells did display a very high tendency to share the same fate (21 out of a total of 24 sibling pairs populating the bin corresponding to the first cell cycle decile; Fisher’s exact test, α = 0.01; Fig. 1E). Rapidly thereafter, however, the cell fate of siblings became completely independent of each other, with the amount of coupling not significantly differing from what would be expected by chance (Fisher’s exact test, α = 0.01; Fig. 1E). Of note is that overall cellular survival within this initial period, as mentioned above, does not significantly differ from other cell cycle periods, indicating that the high amount observed sister cell fate coupling was not a trivial consequence of in- or decreased inactivation during this initial period, and that coupling occurs both in terms of survival and death. Importantly, cell fate coupling itself was not influenced by differences (in measurable attributes) between the respective sibling pairs, as these intersibling differences were similar for sister cells displaying coupled and uncoupled cell fate (Table S1).

Together, these findings illustrate that sister cells, as they originate from their common ancestor, indeed are and behave similar (as illustrated by their shared fate), but that sister cell differentiation (i.e. the individualization of their phenotypic states) rapidly occurs, with siblings apparently forgetting their common ancestry and behaving as independent entities. In addition, the strongly coupled behavior of freshly born sister cells illustrates the potential of our survival-assay to truly reveal stochastic variability between individual cells as opposed to stochastic variability in the distribution of injury (in which case heat would impose randomly uncoupled fates throughout all of the cell cycle bins).

### Sibling individualization also manifests itself during outgrowth

**Figure 2:**
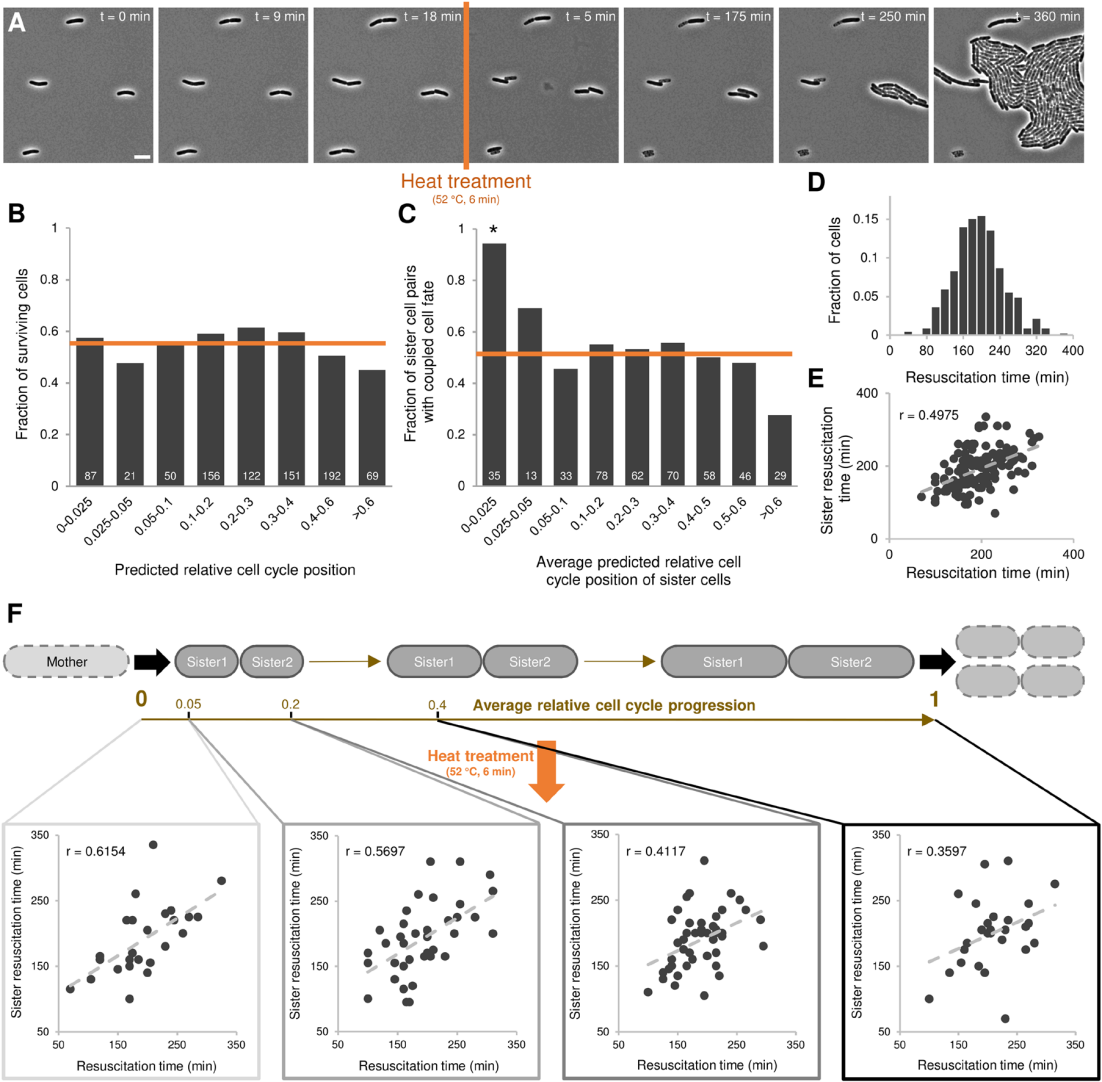
Rapid sibling individualization is also apparent during subsequent outgrowth. (**A**) Representative phase contrast images of a TLFM microscopy image sequence of growing *E. coli* MG1655 cells at indicated times before and after heat treatment (52 °C, 6 min). The scale bar corresponds to 5 µm. (**B**) Fraction of cells surviving the heat treatment (52 °C, 6 min) binned by their predicted relative cell cycle position (n = 848). None of the individual bins were found to differ significantly from the average of all cells (orange line, 55.8%, 95% CI = [52.47%, 59.1%]; Fisher’s exact test, α = 0.01; asterisks indicate significant differences). Indicated in white is the total number of cells that was observed for each bin. (**C**) Fraction of sister cells with coupled cell fate (i.e. both cells either survive or die) is binned by the average predicted relative cell cycle position of the siblings (n = 424 sibling pairs). Indicated in white is the total number of sibling pairs that was observed for each bin. Orange line indicates the amount of coupling that would be expected by chance (50.7%, given the overall level of survival), asterisks indicate significant differences (Fisher’s exact test, α = 0.01). Note that the overall empirical fraction of siblings with shared cell fate (54.5%) is not significantly different from what would be expected by chance (p-value = 0.11). (**D**) Resuscitation time distribution of cells surviving the heat treatment (52 °C, 6 min; n = 473). The time individual surviving cells needed to resume growth was determined and binned to create the resuscitation time distribution. (**E**) Correlation between resuscitation times of surviving sister cell pairs (n = 140 sibling pairs, Pearson’s r = 0.4975, p-value = 1.04 × 10^−9^). (**F**) Evolution of the correlation between surviving siblings’ resuscitation times as these cells progress through their cell cycle. Shown is a schematic of two sibling cells progressing through their corresponding cell cycles. Depending on the timing of the heat treatment (orange arrow), with respect to the relative cell cycle progression of these cells, the correlation of their corresponding resuscitation times (in the case both siblings were able to survive) diminishes as cells advance. Correlations are shown for different cell cycle progression intervals (0–0.05, 0.05–0.2, 0.2–0.4 and 0.4–1, respectively) based on the average predicted relative cell cycle position of surviving siblings (n = 25, 38, 48 and 28 sibling pairs, respectively). Both the strength (respective Pearson’s r-values: 0.6154, 0.5697, 0.4117 and 0.3597, respective bootstrapped 95% CI: [0.3692, 0.8222], [0.3813, 0.7232], [0.1495, 0.6377] and [−0.0364, 0.6947]) and the significance (respective p-values: 4.78 × 10^−4^, 1.9 × 10^−4^, 5.49 × 10^−3^ and 5.83 × 10^−2^) of these correlations decline gradually as surviving siblings progress through their cell cycle before the heat treatment (52 °C, 6 min).

In a final step, we aimed to investigate to what extent the observed individualization, initially observed in terms of survival probability, is reflected in subsequent cellular behavior of surviving cells. To this end, we modified our single-cell level survival-assay in four ways (Fig. 2A). First, we employed wild-type *E. coli* MG1655 cells, given the limited predictive information obtained from visualizing chromosome replication and segregation. Second, we only allowed these cells to grow for one generation before application of the heat shock, given the apparent insignificance of hereditary effects and in order to limit potential interference of neighboring cells on survival and subsequent outgrowth. Third, given that cells grown for only one generation on the agarose pad displayed slightly altered survival tendencies as compared to cells grown for multiple generations, the intensity and duration of the heat treatment (52 °C, 6 min) were adjusted to achieve a similar inactivation (ca. 50%) as before. Fourth, we developed an updated predictive cell cycle progression model, again based on the detailed analysis of growth of unstressed cells in separate control experiments, in order to accommodate cells exposed to a heat shock under these new conditions (Fig. S4).

In agreement with the previous survival-assay, (i) no predisposition factors affecting survival at the single-cell level could be detected (Fig. S5), (ii) sister cells on average displayed independent behavior (54.5% as measured versus 50.7% as expected by chance given the overall frequency of survival (55.8%, out of a total of 848 heat-shocked cells), p-value = 0.11), and (iii) rapid individualization of their phenotypic states could independently be confirmed (Fig. 2B,C). Besides illustrating the robustness and more general applicability of our previous findings, the higher number of cells sampled in early relative cell cycle stages allowed us to zoom in on this early cell cycle period and pinpoint the very early stages after division (within 1/20-1/40 of a cell cycle) as those during which the individualization already occurs (Fig. 2C).

In addition, the absence of the fluorescent nucleoid reporter allowed us to probe the basal activity (i.e. before application of the heat shock) of the general stress (RpoS) and heat shock (RpoH) response sigma factors. To this end, we created fluorescent transcriptional reporter strains of *yiaG* and *ibpA*, genes whose expression is known to be modulated by RpoS and RpoH activity^14, 15^, respectively. The acquired strains (MG1655 *P*_*yiaG*_ *-msfgfp* and *P*_*ibpA*_ *-msfgfp*) were subsequently employed to investigate whether basal sigma factor activity levels affect cellular survival. In line with the apparent stochastic nature of our assay and the rapid individualization of sibling pairs, no significant influence of either basal level of sigma factor activity on survival probability could be detected (Fig. S6A–D). Moreover, the high correlation in sister cell fluorescence intensities for both transcriptional reporters prior to the heat treatment indicated that sigma factor activities likely not underlie or drive the rapid individualization (Fig. S6E,F).

This new experimental setup also allowed us to properly examine the individual resuscitation phase of cells as an additional indicator of their state and behavior after application of the stressor (Fig. 2D). Although no measurable cellular predisposition factors, directly affecting a surviving cell’s resuscitation time, could be identified (Fig. S7), resuscitation times of surviving sister cells were correlated (Fig. 2E). Interestingly, while recently formed (surviving) sister cell pairs displayed highly similar and correlated resuscitation times, this correlation gradually declined as these cells progressed through their cell cycles, indicating that the observed individualization in terms of survival also manifests itself during the subsequent outgrowth of surviving cells (Fig. 2F). In fact, the decrease in resuscitation time correlation of the (surviving) older sibling pairs indicates that the corresponding sister cells differently perceived the heat damage and thus more clearly underscores that they were randomly sharing the same survival fate.

## Discussion

Although many mechanisms underlying the generation of phenotypic heterogeneity within clonal populations have been elucidated, the dynamics by which sister cells differentiate from each other with respect to complex phenotypes such as stress survival have remained unclear. In this work, we challenged clonally growing *E. coli* populations with a semi-lethal heat stress and exploited differences in survival fate as an indicator of stochastic differentiation between sister cells. The superposition of a predictive cell cycle progression model subsequently revealed the rapid individualization of sister cells, both in terms of their cell fate as their subsequent behavior upon survival. Whereas recently formed siblings (i.e. within the first 10% of their cell cycle) were found to behave extremely similar in our setup (as is evident from their strong tendency to share the same fate and, in the case they both survive, display similar resuscitation times), they rapidly displayed independent behavior thereafter (as is evident from their randomized coupling of cell fate). While after the fission event the molecular composition of sister cells is bound to differ instantaneously because of stochastic fluctuations, our results demonstrate the order of magnitude of cell cycle progression necessary to actually achieve a differentiation in phenotype as a result of such stochastic fluctuations.

Remarkably, the timescale of this individualization (within one decile of a cell cycle) is much smaller than that reported for other phenotypic switches^8, 9, 11, 14, 15^. Such previous investigations into the dynamics of cellular differentiation, however, focused solely on clearly defined phenotypic switches, established by relative simple genetic mechanisms that are typically retained over a larger number of generations. In contrast, this study approached the speed of differentiation between sister cells by phenotypically challenging them and illustrating differences in cell fate to function as indicators of stochastic individualization.

A trivial explanation for the observed fate coupling during early cell cycle stages would stem from the erroneous detection of cell division events (i.e. premature detection of cell division before siblings are completely separated). Cytokinesis in *E. coli* is a complex and highly regulated process that can take up to 30% of a cell cycle^16–18^, and thus potentially gives rise to the incorrect identification of cell division timing. In order to prevent such untimely detections of cell division leading to an almost guaranteed coupling of cell fate, each division event was manually validated and its timing was chosen conservatively, ensuring siblings were fully separated at the time of division detection. In addition, the time in between the end of TLFM recordings and the attainment of lethal temperatures in the thermocycler, typically around 1–2 min, should ensure the complete division and separation of cells prematurely identified as divided despite all of the above-mentioned precautions. In addition, this time gap likely leads to a slight underestimation (approx. 5–10% of relative cell cycle progression) of the timescale of fate coupling in our setup. Notably, all the above considerations do not affect the overall independent fate of sibling cells, only the timescale of their apparent individualization.

The cell cycle regression model not only allowed us to accurately predict a cell’s relative point in its cell cycle. Its interpretable structure, consisting of a regression tree with linear models in its leaves, also allows us to assess the relative contribution/importance of each of the included cellular attributes. Given that most nodes in the decision tree focus on a cell’s length increase since birth (ΔL), it is clear that this attribute plays an important role in determining a cell’s relative cell cycle progression. Remarkably, this is in agreement with recent studies identifying the addition of a constant volume each generation (cell width remains constant, thus equating length increase with volume increase) as the predominant cellular strategy to ensure cell size control and homeostasis in bacteria^19, 20^. Our finding that cell cycle progression did not function as a deterministic mechanism underlying cell fate illustrates the relative robustness of ongoing processes and different cell cycle stages to environmental perturbations (cell cycle stage is thus not a sensitizing factor). This contrasts recent findings in *Caulobacter crescentus* which identified cell cycle stage as a critical factor in determining a cell’s fate upon exposure to salt stress^21^. Our setup, however, besides employing a different environmental stressor and employing a more accurate prediction for cell cycle progression (not only based on time since birth), made use of *E. coli* cells growing in LB medium. Under these circumstances, exponentially growing *E. coli* cells harbor multiple active replication forks^22^, potentially obscuring any cell cycle-related effects. In this light, it would be interesting to examine whether cells grown in minimal medium (in which the cell cycle displays a more linear behavior with only one round of chromosome replication per generation without initiation of a second^12^) display a similar cell cycle stage robustness.

In addition, we speculate that our approach, through its general applicability and transferability, should allow the future identification of robust or fragile cell cycle stages in other (unicellular) organisms as well. Subsequent unraveling of the molecular mechanisms hereof should then allow a better general understanding of the impact of various stressors on cellular behavior as well as allow the identification of various cellular survival/defense strategies. Moreover, a similar approach could be used to capture and quantify the varying timescales with which phenotypic heterogeneity is or can be generated in any given microbial population. This, in turn, could lead to an increased understanding of the differentiation dynamics of these populations, and in cases for which the underlying mechanisms are known, interesting insights into how given mechanisms give rise to specific dynamics.

Taken together, our findings highlight (i) the ability of stochastic events to rapidly differentiate highly similar sister cells into phenotypically independent individuals, and (ii) the potential of stressful encounters to expose differential cellular behavior that otherwise remains cryptic in the absence of stress, as many other measurable and complex sister cell characteristics display significant correlations (Fig. S8). Although the underlying mechanisms and evolutionary driving forces of this individualization currently remain elusive, we speculate that similar mechanisms and patterns exist in other clonal populations, including those involved in human diseases such as microbial pathogenesis and cancer, as they too need to cope with complex and potentially lethal environmental fluctuations and signals.

## Methods

### Strain construction and growth conditions

*E. coli* K12 MG1655 was used as a parental strain in this study^23^. In order to create a fluorescent C-terminal HupA-YFP fusion protein allowing dynamic nucleoid visualization^12^, pGBKD-*venus* was first constructed by cutting plasmid pGBKDparSpMT1^24^, with *EcoR*I and *BamH*I, and ligating a *venus*-amplicon^25^, with its start codon replaced by an GSGSGS-linker, created with primers venus_EcoRI_GS_Fw and venus_BamHI_Rev, into the linearized vector. *E. coli* MG1655 *hupA-yfp* was then created by recombineering a *venus-frt-cat-frt* amplicon^26^, obtained from plasmid pGBKD-*venus* by PCR with primers hupA_venus_Fw and hupA_venus_Rev, into the *hupA* gene, replacing its stop codon. Subsequently, the chloramphenicol resistance cassette was excised by transiently equipping this strain with plasmid pCP20 expressing the Flp site-specific recombinase^27^, resulting in the desired *hupA-yfp* strain.

In order to create the fluorescent transcriptional P_*yiaG*_-msfGFP and P_*ibpA*_-msfGFP fusions probing the activity of the general stress (RpoS) and heat shock (RpoH) response sigma factors^28, 29^, a *msfgfp-frt-nptII-frt* amplicon was obtained from plasmid pDHL1029^30^, using primers PyiaG_msfgfp_Fw and PyiaG_msfgfp_Rev, PibpA_msfgfp_Fw and PibpA_msfgfp_Rev, respectively. The respective fluorescent *E. coli* MG1655 transcriptional reporter strains were then created by recombineering this amplicon 5 bp after the stop codon of the gene of interest ensuring co-transcription. To maximize co-translational activity, the gene encoding *msfgfp* was preceded by a strong synthetic ribosome binding site (BBa_B0034; sequence AAAGAGGAGAA^31^). Subsequently, the kanamycin resistance cassette was excised by transiently equipping this strain with plasmid pCP20 expressing the Flp site-specific recombinase^27^, resulting in the desired fluorescent transcriptional reporter strains.

For culturing bacteria, Lysogeny Broth (LB) medium^32, 33^ was used either as a broth, or solid medium after the addition of 2% agarose (for agar pads intended for microscopy). Stationary phase cultures were obtained by growing *E. coli* overnight for approximately 15 hours in LB broth at 37 °C under well aerated conditions (200 rpm on an orbital shaker). The next morning, these cultures were re-inoculated (1/100) into fresh LB medium, grown at 37 °C to an OD600 of 0.2–0.3 and subsequently subjected to time-lapse fluorescence microscopy.

### Primers

Primers used in this study are listed in Table 1.

**Table 1:** Primers used in this study. Primer attachment sites are indicated in bold, linker sequences and artificial ribosome binding sites in *italics*, and restriction sites are underlined.

Name	Sequence (5′-3′)
venus_EcoRI_GS_Fw	AGAATTC *GGCAGCGGCAGCGGCAGC* **GCTAGCAAAGGAGAAGAACT**
venus_BamHI_Rev	AGGATCC **TTATTTGTAGAGCTCATCCATG**
hupA_venus_Fw	CTAACGTACCGGCATTTGTTTCTGGCAAGGCACTGAAAGACGCAGTTAAG**GGCAGCGGCAGCGGCA**
hupA_venus_Rev	AAAAGGGGTGAAACCACCCCTTCGTTAAAACTGTTCACTGCCACGCAATC**GTGTAGGCTGGAGCTGCTTC**
PyiaG_msfgfp_Fw	TTGATTCAAGCCAACCCGGCATTAAGTAAGCAGTTGATGGAATAGACTTT*AAAGAGGAGAA*TACTAGATG**AGTAAAGGTGAAGAACTGTTCACCGG**
PyiaG_msfgfp_Rev	GTTGGAAAACGGTCCTGTCATCAGGACCGTAAACAGCAATAAAGTGGATA**ATTCCGGGGATCCGTCGACC**
PibpA_msfgfp_Fw	GTGATTCCGGAAGCGAAAAAACCGCGCCGTATCGAAATCAACTAATTCCC*AAAGAGGAGAA*TACTAGATG**AGTAAAGGTGAAGAACTGTTCACCGG**
PibpA_msfgfp_Rev	CCTGACGGCGAGCATGGAGATGTCAGGCCGCGCCAGGCGGCCTTAGGGAATTAGTTGATT**ATTCCGGGGATCCGTCGACC**

### Time lapse fluorescence microscopy (TLFM)

For TLFM, cell suspensions were diluted appropriately in LB, transferred to LB agarose (2%) pads placed on a microscopy slide, and mounted with a cover glass. A Gene Frame (Thermo Scientific) was used to hold the cover glass on the microscopy slide. TLFM of user-selected positions was performed with a temperature controlled (37 °C; Okolab, Ottaviano, Italy) Ti-Eclipse inverted microscope (Nikon, Champigny-sur-Marne, France) equipped with a 60x objective, a TI-CT-E motorized condenser, a YFP filter (Ex 500/24, DM 520, Em 542/27), a GFP filter (Ex 472/30, Dm 495, Em 520/35) and a CoolSnap HQ2 FireWire CCD-camera. Phase contrast images were acquired every 30–60 seconds, YFP images every 4 min using NIS-Elements microscope control software (Nikon). The resulting images were further handled with open source software ImageJ.

### Image analysis and quantitative analysis of single-cell level growth

Characteristics (e.g. length, area, fluorescence) of individual cells growing in/into microcolonies (and of the microcolonies themselves) were acquired using the MicrobeTracker software^34^. In order to obtain robust results, manual curation was necessary to improve automatic segmentation and tracking. During manual curation, special care was taken to prevent premature detection of cell division events by manually validating and, if necessary, correcting each division event. Overall, division events were detected conservatively in order to ensure complete separation of siblings at the time of division (i.e. when a small light grey band became apparent between dividing sister cells in the phase contrast image). The data generated by this analysis was fed into a relational database enabling its subsequent transformation (e.g. calculation of certain cellular characteristics (growth rate), establishment of genealogical relationships between cells) and mining.

Growth rates of microcolonies were determined by exponential fits of microcolony area over time. Given the relative constancy of cell width during cell cycle progression, cell length was employed to quantify to quantify cellular growth of individual cells. Growth rates of individual cells were determined by exponential fits of cell length over time. Instantaneous growth rates were determined by exponential fits of cell length over a time window of 7 minutes (typically the last 7 min of growth before heat treatment). For recently divided cells (within this 7 min time window), half of the length of the mother cell was used with a correction for asymmetric division events. Cell age was inferred from old pole generations as introduced by Stewart *et al*.^35^.

### Heat treatment

For heat shock experiments, the same cells were microscopically examined before and after heat treatment. To accomplish this, cells were first mounted on a microscopy slide as described above, allowed to grow for approximately 4 generations (MG1655 *hupA-yfp*; microcolonies consisting of 8–23 cells) or for exactly 1 generation (MG1655, MG1655 *P*_*yiaG*_ *-msfgfp* and MG1655 *P*_*ibpA*_ *-msfgfp*; 2 sibling cells; cells having undergone no or two cell divisions were excluded from the analysis) while their spatial coordinates on the slide were noted. Please note that the size of the microcolonies was chosen so to limit nutrient limitation and positional effects (i.e. localization within the microcolony) from trivially affecting cellular behavior, but at the same time allows for potential hereditary effects to be detected.

Subsequently, the slide as a whole was subjected to a heat shock (49 °C, 20 min for MG1655 *hupA-yfp* cells; 52 °C, 6 min for MG1655, MG1655 *P*_*yiaG*_ *-msfgfp* and MG1655 *P*_*ibpA*_ *-msfgfp* cells; heat shock durations and intensities were chosen so to inactivate approximately half of the cellular population) by taping the slide to the lid of a thermocycler (Westburg, Leusden, the Netherlands), after which the spatial coordinates were used to trace back and microscopically follow-up the same cells on the heat-treated slide. For each of the abovementioned experiments, control experiments were also performed during which the cells were allowed to grow without application of a heat shock. These control experiments generated the necessary training data for constructing the predictive cell cycle model.

### Construction and validation of a predictive relative cell cycle progression model

In our survival-assay, exponentially growing *E. coli* cells are exposed to a heat treatment at a given point in time. Due to its stochastic nature and dependence on previous generations, cell cycle progression will vary between each of the individual cells present at the time of heat shock. To accurately predict a cell’s relative cell cycle position at the moment of heat shock, a regression model was constructed. Construction of this model, which allows the quantification of relative cell cycle progression as a number between 0 and 1 (which correspond to a cell’s birth and division, respectively), was necessary since it is impossible to exactly determine cell cycle progression directly. To this end, a training dataset was created based on the detailed analysis of the growth of unstressed cells in separate control experiments (n = 635 for MG1655 *hupA-yfp* and n = 453 for MG1655 wild-type cells), i.e. cells that were observed from birth to division. From these training cells, a relative point in their cell cycle was sampled and several cellular features (length at birth, time since birth, length increase since birth, growth rate, increase in cellular DNA content, cellular DNA content) were recorded. Based on this training dataset, a model tree was learned^36^ which consists of a regression tree with linear models in the leaves. The split criterion minimizes the mean squared error of the linear models of each subsequent leaf. To prevent overfitting, a minimal amount of 25 cells per leaf was imposed. The model tree was implemented based on the regression tree of the scikit-learn python package^37^. Given the different experimental setup used for both strains (4 generations vs. 1 generation of growth on LB agarose pads under the microscope before heat treatment), dedicated cell cycle models were constructed and trained for MG1655 *hupA-yfp* and MG1655 wild-type cells. The current model trees were chosen for their simplicity and relative interpretability among similarly performing models like random forest and boosting methods. Simpler methods such as linear regression were also tested but did not perform as well. The depth of the model trees was set to 3 on the basis of internal cross-validation.

Extremely large cells (i.e. filamentous cells of which the cell length >11 µm) were excluded from the training dataset and in the prediction of relative cell cycle progression due to their tendency to produce aberrant results (in constructing the regression models and in predicting a cell’s relative cell cycle progression, respectively).

Importantly, the short cell cycle period during which the individualization process was observed is, at least with respect to time, not smaller than our sampling frequency (every 30 s with an average doubling time of 23.44 min, based on the analysis of unstressed cells, which equates to sampling approximately 47 times, or every 2.16%, for the average cell cycle), ensuring the reported timing of individualization is not an artefact of an insufficiently high sampling frequency.

### Determination of viability and resuscitation time measurements

Cellular viability (i.e. the relative number of cells surviving the heat sock) was determined by TLFM. Cells that could be observed to grow and divide within a 6 h and 10 h time frame after heat treatment, for MG1655 *hupA-yfp* and MG1655 wild-type cells respectively, were scored as surviving cells.

Cell meshes generated by the MicrobeTracker program were used to determine resuscitation times of individual cells, as described previously^38^. Since bacterial cells typically only elongate in the longitudinal direction, resuscitation times were measured by looking at the length increase of individual cells over time. First, an initial length was calculated as the mean of the first three measurements for each individual cell. The length of that cell in the subsequent frames was then compared to this initial length, and the resuscitation time was defined as the time corresponding to the frame where cell length had increased over 10% compared to its initial length, plus the time between the end of the heat treatment and the beginning of microscopy recording (typically around 5 min). This 10% increase in initial length was taken as a threshold to prevent random measurement fluctuations from influencing the results and ensure that only resuscitation times of cells that had fully committed to growth were measured. In addition, only resuscitation times of surviving cells were measured, i.e. cells that subsequently committed to growth and division.

### Examination of sibling cell fate coupling

Given that, overall, each individual cell’s chance of survival follows a Bernouilli distribution with success parameter p = 0.454 and p = 0.558, for MG1655 *hupA-yfp* and MG1655 wild-type cells respectively, the theoretic probability of siblings sharing the same fate, under the null hypotheses that siblings behave independently of each other, can be calculated by p² + (1-p)². This equates to 0.504 and 0.507, for the 273 pairs of MG1655 *hupA-yfp* and 424 pairs of MG1655 wild-type cells respectively, which can directly be compared to the observed frequency of 0.497 and 0.545 using a binomial test. This test yields p-values of 0.79 and 0.11, indicating that for both datasets, the null hypothesis that siblings display independent behavior in terms of cell fate cannot be rejected.

### Data availability

The datasets generated during and/or analyzed during the current study are available from the authors on reasonable request.

## Electronic supplementary material

Supplemental information

## References

[1] Avery SV (2006). Microbial cell individuality and the underlying sources of heterogeneity. Nature reviews. Microbiology.

[2] Elowitz MB, Levine AJ, Siggia ED, Swain PS (2002). Stochastic gene expression in a single cell. Science.

[3] Ozbudak EM, Thattai M, Kurtser I, Grossman AD, van Oudenaarden A (2002). Regulation of noise in the expression of a single gene. Nature genetics.

[4] Ackermann M (2015). A functional perspective on phenotypic heterogeneity in microorganisms. Nature reviews. Microbiology.

[5] West SA, Cooper GA (2016). Division of labour in microorganisms: an evolutionary perspective. Nature reviews. Microbiology.

[6] Kussell E, Leibler S (2005). Phenotypic diversity, population growth, and information in fluctuating environments. Science.

[7] Casadesus J, Low DA (2013). Programmed heterogeneity: epigenetic mechanisms in bacteria. The Journal of biological chemistry.

[8] Norman TM, Lord ND, Paulsson J, Losick R (2013). Memory and modularity in cell-fate decision making. Nature.

[9] Robert L (2010). Pre-dispositions and epigenetic inheritance in the Escherichia coli lactose operon bistable switch. Molecular systems biology.

[10] Suel GM, Kulkarni RP, Dworkin J, Garcia-Ojalvo J, Elowitz MB (2007). Tunability and noise dependence in differentiation dynamics. Science.

[11] Norman TM, Lord ND, Paulsson J, Losick R (2015). Stochastic Switching of Cell Fate in Microbes. Annual review of microbiology.

[12] Fisher JK (2013). Four-dimensional imaging of E. coli nucleoid organization and dynamics in living cells. Cell.

[13] Smelt JP, Brul S (2014). Thermal inactivation of microorganisms. Critical reviews in food science and nutrition.

[14] Veening JW (2008). Bet-hedging and epigenetic inheritance in bacterial cell development. Proceedings of the National Academy of Sciences of the United States of America.

[15] Ni M (2012). Pre-disposition and epigenetics govern variation in bacterial survival upon stress. PLoS genetics.

[16] Buss J (2015). A multi-layered protein network stabilizes the Escherichia coli FtsZ-ring and modulates constriction dynamics. PLoS genetics.

[17] Coltharp C, Buss J, Plumer TM, Xiao J (2016). Defining the rate-limiting processes of bacterial cytokinesis. Proceedings of the National Academy of Sciences of the United States of America.

[18] Gray, A. N. *et al*. Coordination of peptidoglycan synthesis and outer membrane constriction during Escherichia coli cell division. *eLife* **4**, 10.7554/eLife.07118 (2015). 10.7554/eLife.07118 PMC4458516 25951518

[19] Campos M (2014). A constant size extension drives bacterial cell size homeostasis. Cell.

[20] Taheri-Araghi S (2015). Cell-size control and homeostasis in bacteria. Current biology: CB.

[21] Mathis R, Ackermann M (2016). Response of single bacterial cells to stress gives rise to complex history dependence at the population level. Proceedings of the National Academy of Sciences of the United States of America.

[22] Bipatnath M, Dennis PP, Bremer H (1998). Initiation and velocity of chromosome replication in Escherichia coli B/r and K-12. Journal of bacteriology.

[23] Blattner FR (1997). The complete genome sequence of Escherichia coli K-12. Science.

[24] Espeli O, Mercier R, Boccard F (2008). DNA dynamics vary according to macrodomain topography in the E. coli chromosome. Molecular microbiology.

[25] Nagai T (2002). A variant of yellow fluorescent protein with fast and efficient maturation for cell-biological applications. Nature biotechnology.

[26] Datsenko KA, Wanner BL (2000). One-step inactivation of chromosomal genes in Escherichia coli K-12 using PCR products. Proceedings of the National Academy of Sciences of the United States of America.

[27] Cherepanov PP, Wackernagel W (1995). Gene disruption in Escherichia coli: TcR and KmR cassettes with the option of Flp-catalyzed excision of the antibiotic-resistance determinant. Gene.

[28] Fontaine F, Stewart EJ, Lindner AB, Taddei F (2008). Mutations in two global regulators lower individual mortality in Escherichia coli. Molecular microbiology.

[29] Richmond CS, Glasner JD, Mau R, Jin H, Blattner FR (1999). Genome-wide expression profiling in Escherichia coli K-12. Nucleic acids research.

[30] Ke N, Landgraf D, Paulsson J, Berkmen M (2016). Visualization of Periplasmic and Cytoplasmic Proteins with a Self-Labeling Protein Tag. Journal of bacteriology.

[31] Elowitz MB, Leibler S (2000). A synthetic oscillatory network of transcriptional regulators. Nature.

[32] Miller, J. H. A short course in bacterial genetics: a laboratory manual and handbook for Escherichia coli and related bacteria. (Cold Spring Harbor Laboratory Press, 1992).

[33] Bertani G (2004). Lysogeny at mid-twentieth century: P1, P2, and other experimental systems. Journal of bacteriology.

[34] Sliusarenko O, Heinritz J, Emonet T, Jacobs-Wagner C (2011). High-throughput, subpixel precision analysis of bacterial morphogenesis and intracellular spatio-temporal dynamics. Molecular microbiology.

[35] Stewart EJ, Madden R, Paul G, Taddei F (2005). Aging and death in an organism that reproduces by morphologically symmetric division. PLoS biology.

[36] Quinlan, J. R. in 5th Australian joint conference on artificial intelligence. 343–348.

[37] Pedregosa F (2011). Scikit-learn: Machine Learning in Python. Journal of Machine Learning Research.

[38] Govers SK, Dutre P, Aertsen A (2014). *In vivo* disassembly and reassembly of protein aggregates in Escherichia coli. Journal of bacteriology.

