# Supplementary material for: Rapid phenotypic individualization of bacterial sister cells
Source: Sci Rep. 2017 Aug 16;7:8473. doi: 10.1038/s41598-017-08660-0 (PMC5559607; doi:10.1038/s41598-017-08660-0)
Supplement: Supplementary file 1 — Supplemental information [file 41598_2017_8660_MOESM1_ESM.pdf]

Supplemental material: "Rapid phenotypic individualization of bacterial sister cells"

Sander K. Govers, Antoine Adam, Hendrik Blockeel and Abram Aertsen

Supplemental text: survival is not governed by epigenetic determinants nor by predispositions

As sister cells did not display a tendency to share the same fate in our assay, we set forward to further investigate the potential absence of epigenetic factors and predisposition factors governing survival in our assay.

In a first step, we investigated whether the observed intermicrocolony variation could be caused by differentiation events occurring before cells were placed on the agarose pad (instead of being the consequence of random stochasticity). On average, microcolonies appeared to behave similarly in terms of cellular survival and strong deviations from the average fraction of surviving cells within a microcolony were limited (Fig. S1A, coefficient of variation = 34.9 %). To further investigate this, we randomly resampled our data to generate randomized trees in which progeny (emerging from the original ancestors) is randomly reassigned, and compared the obtained distributions of the proportion of surviving cells within microcolonies to the empirical distribution (Figure S1B). If the abovementioned differentiation events were to exist and govern cell fate in our experimental setup, many of these distributions are expected to differ significantly from the empirical distribution. However, none of the distributions obtained from the randomly resampled populations were found to significantly differ from the empirical one (Kolmogorov-Smirnov (K-S) test, $\alpha = 0.05$; Figure S1B), suggesting the latter is not significantly affected by differentiation events (that are then inherited over multiple generations) occurring before monitoring of cellular growth.

Given the limited number of monitored microcolonies (n = 29), we set forward to examine these findings further using a set of complementary approaches. We first employed bootstrapping to assess the potential variability in average microcolony level survival. To this end, we generated 10 000 bootstrap samples by sampling with replacement from our original sample containing the survival frequency of the monitored microcolonies and used the obtained distribution of the means to calculate the 95 % confidence interval of average microcolony level survival. Using this method, a 95 % confidence interval of [0.3996, 0.5142] was obtained, indicating average survival in this experimental setup indeed lies just below half of the cells within a given microcolony. In addition, Shapiro-Wilk and Anderson-Darling tests on the empirical distribution indicated that the fraction of surviving cells within a microcolony likely is normally distributed (p-value = 2.52×10^{-1} and 1.17×10^{-1}, respectively), a finding strengthened by the quantile-quantile plot (Figure S1C). Together, these findings again suggest that average microcolony level survival is not significantly affected by epigenetically inherited factors predating the beginning of TLFM recording, as these would give rise to highly variable, non-normal microcolony survival frequencies.

We also examined whether any of the variability in survival frequency could be attributed to a measurable property of the microcolonies. To this end, we looked for potential correlations as these would allow us to identify possible determinants of increased or decreased survival frequencies. However, only microcolony growth rate, expressed in terms of area increase, appeared to marginally correlate with average survival within microcolonies (Pearson's r = 0.3932, p-value = 3.77×10^{-2}), with slower microcolony growth rates leading to slightly increased cellular survival (Figure S1D). Other attributes, such as the total number of cells or the area of the microcolony, did not correlate with

cellular survival frequency, altogether indicating that no major survival determinant was active on the microcolony level (Figure S1D).

Besides variation between microcolonies, we also investigated whether epigenetically inherited determinants of cell fate (leading to increased or decreased chance of survival) were active within individual microcolonies. If such factors were to exist and be decisive for the outcome of our assay, surviving (and non-surviving) cells should be more closely related to each other than expected by chance (random distribution). Therefore, we first looked at the correlation between the survival rate of the progeny of second and third generation sister cells (later generations of siblings displayed too little descendants to perform any relevant analysis) (Figure S1E). In both cases, no significant correlation was found between the fraction of surviving progeny of sister cells (Pearson's r = 0.2466 and p-value = 1.64×10^{-1} for second generation sister cells, Pearson's r = 0.0436 and p-value = 3.76×10^{-1} for third generation sister cells; Figure S1F). Similarly as before, we used a bootstrapping approach to generate 95 % confidence intervals for these Pearson correlation coefficients. Confidence intervals of [-0.1846, 0.5824] and [-0.3122, 0.2179] were obtained for correlation coefficients comparing surviving progeny of second and third generation sister cells respectively. Together, these findings indicate that progeny survival frequency of earlier generation sister cells displayed limited to no correlation, in turn suggesting survival within microcolonies is not dominated by epigenetic factors. Although the lack of correlation does not completely rule out the existence of any epigenetically inherited cell fate determinants, it does indicate that they do not dominate survival in our experimental setup and that most epigenetic memory inherited from parental cells, with respect to survival, decays over a timescale of less than two generations. Moreover, a direct pairwise comparison of the average survival of the

descendants of second and third generation sister cells revealed no significant differences between progeny groups (Student's t-test, $\alpha = 0.01$; Figure S1G), additionally indicating that (average) survival was indeed largely unaffected by epigenetically inherited factors.

Although the above already suggests that survival in our assay is indeed randomly distributed (with a given frequency) and not dominated by epigenetic inheritance, we also performed a parsimony reconstruction of ancestral states. This reconstruction estimates cell fate history by minimizing the total number of changes in fate decisions occurring in previous generations [1], and allowed us to assess the appearance of mother cells giving rise to mostly dying or surviving offspring (Figure S1H). This analysis revealed that although survival or death in some cases can be traced back for multiple generations (meaning that related cells such as siblings or cousins have identical fates), in most others, in line with the apparent independent behavior of siblings, cell fate appears to be completely randomly distributed within the microcolony lineage tree (Figure S1I).

Since epigenetic inheritance did not appear to influence the outcome of our survival-assay, we subsequently set forward to assess the role of possible predisposition factors (affecting a cell fate) at the level of the individual cell (Fig. S2). On average, no measurable predisposition factor, increasing or decreasing an individual cell's chances of survival, could be identified (Fig. S2A). Comparing distributions of these attributes for surviving and non-surviving cells elucidated no significant differences (K-S test, $\alpha = 0.01$; Fig. S2A). Moreover, binning the cells by a given cellular attribute and examining survival frequency of these bins failed to identify any subgroups exhibiting a significantly altered survival phenotype (Fisher's exact test, $\alpha = 0.01$, Fig. S2B), altogether indicating the absence of

direct cell fate predisposition factors. Only instantaneous growth rate, expressed in terms of length increase during the last 7 min of growth, appeared able to slightly (but not significantly, Fisher's exact test, $\alpha = 0.01$) influence a cell's fate, with, similar as to what was observed on the microcolony level (Fig. S1D), slower growth rates leading to increased chances of survival (Fig. S2B). This effect, however, appeared rather small and would in no way be able to explain all the observed variability in survival.

Taken together, no direct predisposition factors nor epigenetically inherited determinants appear to affect cell fate in our survival-assay, suggesting that fate itself is determined stochastically. We do not, however, exclude the possibility that other factors, not measured or captured by our experimental setup, are able to influence cellular survival. Nevertheless, if such factors were to exist, they would need to globally affect cellular survival in order to exert a significant effect and be detected in our experimental setup (given the 45.4 % of overall survival). For example, very small subpopulations of cells with higher survival probabilities, analogous to persisters in the context of antibiotic exposure, could be present in our experimental setup. However, their relative contribution to overall survival frequency, given the large number of additional surviving cells, would be limited and thus render them of negligible importance here. In addition, these factors would have to be highly variable on short timescales (within one cell division, as is exemplified by the apparent independent behavior of siblings), without leading to any measurable variations in cellular physiology.

Supplemental methods

Normality testing

Shapiro-Wilk and Anderson-Darling tests for normality were performed in MATLAB. Quantile-quantile plot illustrating potential normality was generated using the built-in MATLAB qqplot function.

Bootstrapping

Bootstrapping in order to generate 95 % confidence intervals of parameters of interest (mean, correlation coefficient) was performed in MATLAB using the built-in *bootci* function. The function calculates the desired confidence interval based on a specified number of bootstrap samples (n = 10 000) generated by sampling with replacement from the original sample.

Random resampling

Random resampling of the MG1655 *hupA-yfp* dataset was performed by randomly swapping 2 cells (and their corresponding descendants) between microcolonies, and repeating this random swap 10 000 times to generate one randomized dataset. To prevent the generation of undesirable long and short randomized microcolonies (within one randomized dataset), cells from the first generation were excluded from the

randomization process and only cells from the same generation were swapped. In total, this process was repeated 200 times, thus generating 200 randomized datasets.

Parsimony reconstruction

Parsimony is a method often used in the construction of evolutionary trees which minimizes the number of evolutionary changes required to reconstruct a current situation[2]. The parsimony reconstruction in this study was performed on all microcolonies within the MG1655 *hupA-yfp* dataset which includes 445 cells that were present at the moment of heat shock, each having their own individual fate (survive or die). A parsimony map of cell fate within these lineages was created by gradually assessing survival tendencies of siblings/cousins/second cousins/etc. If all of these related individuals display the same fate (i.e. all cells either survive or die), their corresponding parent/grandparent/great grandparents/etc. is considered to display a unique parsimonious state and its corresponding line in the lineage tree is colored accordingly.

Supplemental material references

1 Veening, J. W. *et al.* Bet-hedging and epigenetic inheritance in bacterial cell development. *Proceedings of the National Academy of Sciences of the United States of America* **105**, 4393-4398, doi:10.1073/pnas.0700463105 (2008).

2 Pagel, M. Inferring the historical patterns of biological evolution. *Nature* **401**, 877-884, doi:10.1038/44766 (1999).

Supplemental figure legends

Figure S1. Survival is not governed by epigenetic determinants

(A) Distribution of the fraction of surviving cells within a microcolony (n = 29). (B) Histogram of p-values of K-S tests for comparison of empirical proportions of surviving cells in the microcolonies and proportions obtained by data resampling (200 sets of resampled data). (C) Quantile-quantile plot of the fraction of surviving cells within a microcolony versus the theoretical quantile values for a normal distribution. (D) Correlation between the fraction of surviving cells (within a microcolony) and the microcolony growth rate, the number of cells and the microcolony area (n = 29). All correlations are negative, but only influence the fraction of survivors to a limited extent (p-value = 3.77×10^{-2}, 2.78×10^{-1} and 3.20×10^{-1}, 95 % confidence interval (CI) of Pearson's determined by bootstrapping r = [-0.6978,0.0271], [-0.4833,0.1852] and [-0.4092,0.1747], respectively). (E) Diagram of a typical genealogical tree within our survival-assay, with pairs of sister cells indicated (blue: second generation, purple: third generation). The horizontal orange line corresponds to the moment of heat treatment (49 °C, 20 min). Surviving cells are indicated by a green line, non-surviving cells by a red line. (F) Correlation between the survival rate of the progeny of second (blue) and third (purple) generation sister cells (Pearson's r = 0.2466 and p-value = 1.64×10^{-1} for second generation sister cells (n = 29), Pearson's r = 0.0436 and p-value = 3.76×10^{-1} for third generation sister cells (n = 58)). (G) Histogram of p-values of Student's t-test directly comparing survival rate of second (blue) and third (purple) generation sister cells. (H) Parsimony mapping of cell fate onto the lineages of all observed microcolonies ($n_{microcolonies}$ = 29, n_{cells} = 821 cells). Every end point in the tree represents one cell exposed

to the heat treatment; green tips: surviving cells, red tips: non-surviving cells. Colored lines of previous generations correspond to cells with a unique parsimonious state (green: all offspring survives, red: all offspring dies). (I) Histogram displaying the number of generations similar cell fate can be traced back (i.e. 0: sister cells have a different fate, 1: sister cells share the same fate but the fate of at least one of their cousins differs, 2: 4 cousins share the same fate but the fate at least one of their second cousins differs, etc.). ND = no cells could be detected.

Figure S2. Survival-assay is devoid of direct predisposition factors at the single-cell level.

On average, none of the MG1655 *hupA-yfp* attributes was found to significantly differ between surviving and non-surviving cells (Student's t-test, respective p-values: 9.06×10^{-1}, 8.42×10^{-1}, 7.54×10^{-1}, 9.52×10^{-1}, 2.17×10^{-1}, 8.74×10^{-1}, 5.72×10^{-1}, 2.76×10^{-1}, 6.22×10^{-1} and 8.74×10^{-1}). (A) Distributions of cellular attributes of surviving and non-surviving cells (depicted in green and red with n = 193 and n = 232, respectively). No significant differences could be identified (K-S test, $\alpha = 0.01$). (B) In all figures, the fraction of cells surviving the heat treatment (49 °C, 20 min) is binned by a measurable cellular attribute. Individual bins were compared to the average of all cells (orange line; Fisher's exact test, $\alpha = 0.01$), and none were found to significantly differ. Indicated in white is the total number of cells that was observed for each bin. Please note that since Fisher's exact test takes the number of observations per bin into account, bins containing a lower number of cells are less likely to differ significantly from the average of all cells (45.4 %, 95 % CI = [40.7 %, 50.2 %], n = 425).

Figure S3. Detailed structure of the predictive cell cycle progression model for MG1655 *hupA-yfp* cells.

(A) Schematic representation of our survival-assay for an individual cell. As cells grow, many measurable cellular attributes (Lb = length at birth, Δt = time since birth, ΔL = length increase since birth, GR = growth rate, ΔF = increase in cellular DNA content, F = cellular DNA content) can be employed to predict a cell's relative position in its cell cycle at the moment of heat treatment (x). (B) The model itself consists of 7 linear models (LM) preceded by a regression tree. Green arrows indicate a positive answer, red arrows a negative answer. Apostrophes indicate normalized attributes (attr' = (attr–min(attr))/(max(attr)–min(attr)).

Figure S4. Construction and validation of the predictive cell cycle progression model for wild-type MG1655 cells.

(A) Schematic representation of the model structure which consists of 8 linear models (LM) preceded by a regression tree. Green arrows indicate a positive answer, red arrows a negative answer. Apostrophes indicate normalized attributes (attr' = (attr–min(attr))/(max(attr)–min(attr)). (B) Validation of the constructed cell cycle model. The performance of the model was assessed by 10-fold internal cross-validation (n = 435; R^2 = 0.8701, p-value = 2.76×10^{-164}, RMSE = 0.088). The bisector is shown as a dashed orange line. Inset displays the evolution of the R^2 value, calculated by examining the correlation between predicted and actual relative cell cycle progression per independent decile.

Figure S5. The adjusted survival-assay is also devoid of direct predisposition factors at the single-cell level.

(A) Distributions of MG1655 attributes of surviving and non-surviving cells (depicted in green and red with n = 473 and n = 375, respectively). No significant differences could be identified (K-S test, $\alpha = 0.01$). (B) In all figures, the fraction of cells surviving the heat treatment (52 °C, 6 min) is binned by a measurable cellular attribute. Individual bins were compared to the average of all cells (orange line; Fisher's exact test, $\alpha = 0.01$), and none were found to significantly differ. Please note that since Fisher's exact test takes the number of observations per bin into account, bins containing a lower number of cells are less likely to differ significantly from the average of all cells (55.8 %, 95 % CI = [52.47 %, 59.1 %], n = 848). Indicated in white is the total number of cells that was observed for each bin.

Figure S6. Basal RpoS and RpoH activity do not significantly influence individual cell fate.

(A-B) The average cellular fluorescence before heat treatment (52 °C, 6 min) of (A) MG1655 P_{yiaG}-*msfgfp* cells (n = 130) and (B) MG1655 P_{ibpA}-*msfgfp* cells (n = 112), calculated for surviving and non-surviving cells. The measured fluorescence intensities are indicative for the activity of the general stress (RpoS) and heat shock (RpoH) response sigma factor, respectively. For both strains, no significant difference in fluorescence between the two cellular groups (surviving vs. non-surviving) could be detected (Student's t-test, p-values are 2.98×10^{-1} and 3.59×10^{-1}, respectively). Error bars indicate the standard error of the mean. 95 % CI for the difference between surviving and non-

surviving cells is [-7.5,23.8] and [-34.3,12.4], respectively. (C-D) Distribution of average cellular fluorescence of (C) MG1655 P_{yiaG}-*msfgfp* and (D) MG1655 P_{ibpA}-*msfgfp* surviving and non-surviving cells (depicted in green and red, respectively). No significant differences between the distributions of surviving and non-surviving cells could be detected (K-S test, $\alpha = 0.01$). (E-F) Correlation between the average cellular fluorescence of (E) MG1655 P_{yiaG}-*msfgfp* and (F) MG1655 P_{ibpA}-*msfgfp* sibling pairs prior to the heat shock (n = 65 and 56 sibling pairs, respectively). For both transcriptional reporters, a strong significant correlation could be detected (Pearson's r = 0.7998, p-value = 2.58×10^{-15} and Pearson's r = 0.8062, p-value = 1.16×10^{-13}, respectively). The bisector is shown as a dashed orange line.

Figure S7. Resuscitation times are not influenced by measurable cellular characteristics.

Correlation between cellular characteristics before and resuscitation times after heat treatment (52 °C, 6 min) of individual *E. coli* MG1655 cells (n = 848). None of the measured/predicted characteristics (cell length, time since birth, length increase since birth, growth rate, instantaneous growth rate, cell length at birth and predicted relative cell cycle position) strongly affected resuscitation times (respective p-values: 3.92×10^{-1}, 3.09×10^{-1}, 1.07×10^{-1}, 3.54×10^{-2}, 4.76×10^{-3}, 1.22×10^{-1} and 1.50×10^{-1}, and respective 95 % CI of Pearson's r (determined by bootstrapping): [-0.1049,0.0941], [-0.1130,0.0666], [-0.1456,0.0350], [-0.1597,0.0202], [-0.1896,-0.0078], [-0.0511,0.1642] and [-0.1394,0.0440]).

Figure S8. Sibling attributes not related to survival and resuscitation are correlated.

(A-D) Correlation between the (A) length at birth (Pearson's r = 0.8154, p-value = 1.00×10^{-277}), (B) growth rate (Pearson's r = 0.6891, p-value = 1.55×10^{-166}), (C) doubling time (Pearson's r = 0.5526, p-value = 3.26×10^{-81}), and (D) length increase between birth and division (Pearson's r = 0.5101, p-value = 3.37×10^{-77}) of sibling pairs (n ≥ 1157 sibling pairs). For these analyses, data from unstressed MG1655 *hupA-yfp* cells growing in/into microcolonies (third and fourth generation; initially used for the construction of the predictive cell cycle progression model) was used. For (A) and (D) extremely large cells (> 10 μm) were excluded from the analysis due to their tendency to give aberrant results. The bisector is shown as a dashed orange line.

Supplemental table

Table S1. Intersibling differences between sister cells displaying coupled and uncoupled cell fate.

Cellular attribute	*Average*		*Stdev*		*p-value (Student's T-test)*
	Coupled	*Uncoupled*	*Coupled*	*Uncoupled*	
Age	1.86	1.64	0.85	0.80	0.07
Length at birth (μm)	0.5687	0.4653	0.5357	0.3773	0.14
Growth rate (1/min)	0.0064	0.0047	0.0128	0.0053	0.23
Instantaneous growth rate (1/min)	0.0085	0.0078	0.0114	0.0101	0.64
Cellular DNA content (A.U.)	224.26	326.81	242.02	470.42	0.06
Predictive relative cell cycle position	0.053	0.050	0.056	0.050	0.62

Supplemental figures

Figure S1

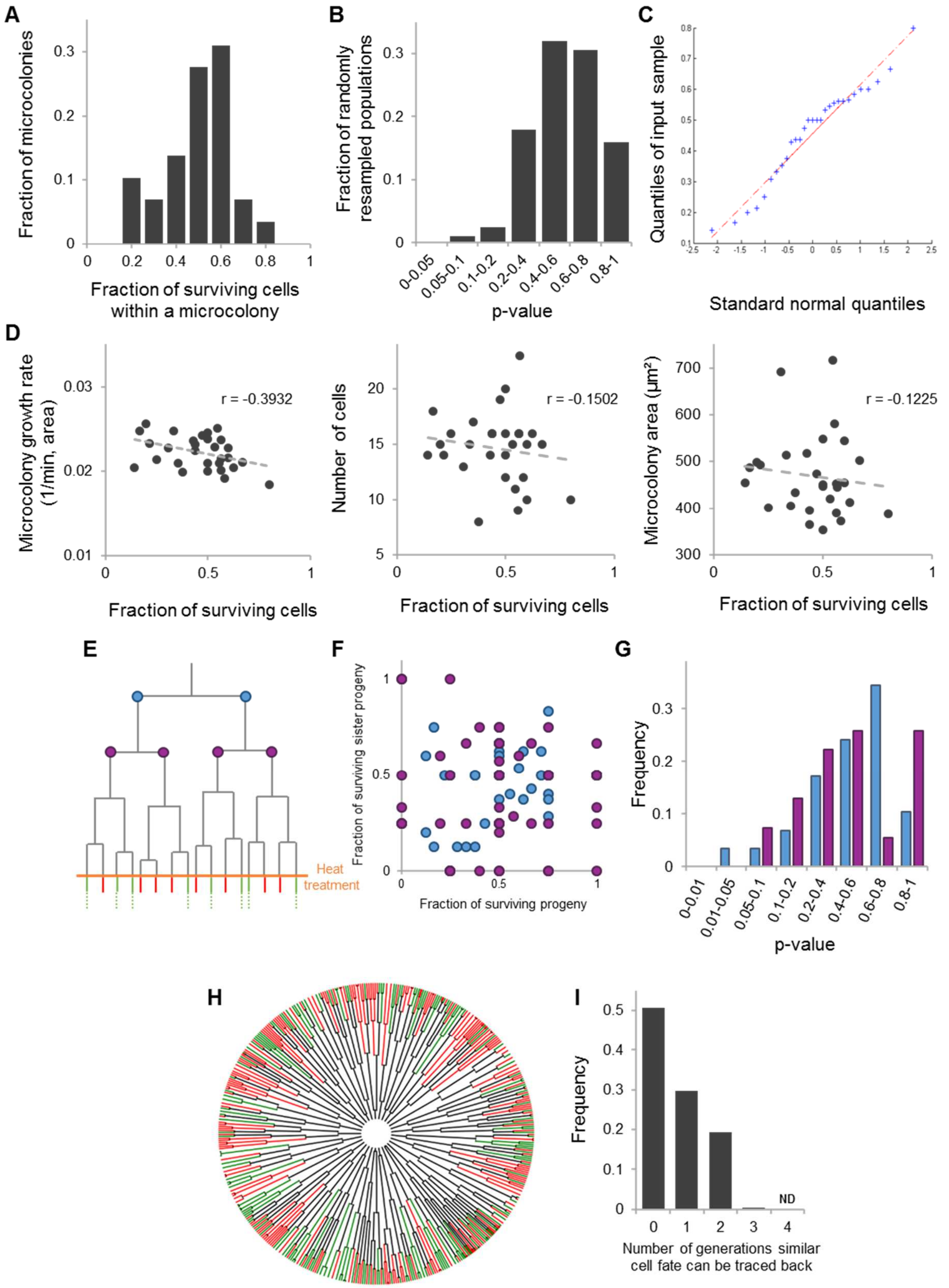

Figure S2

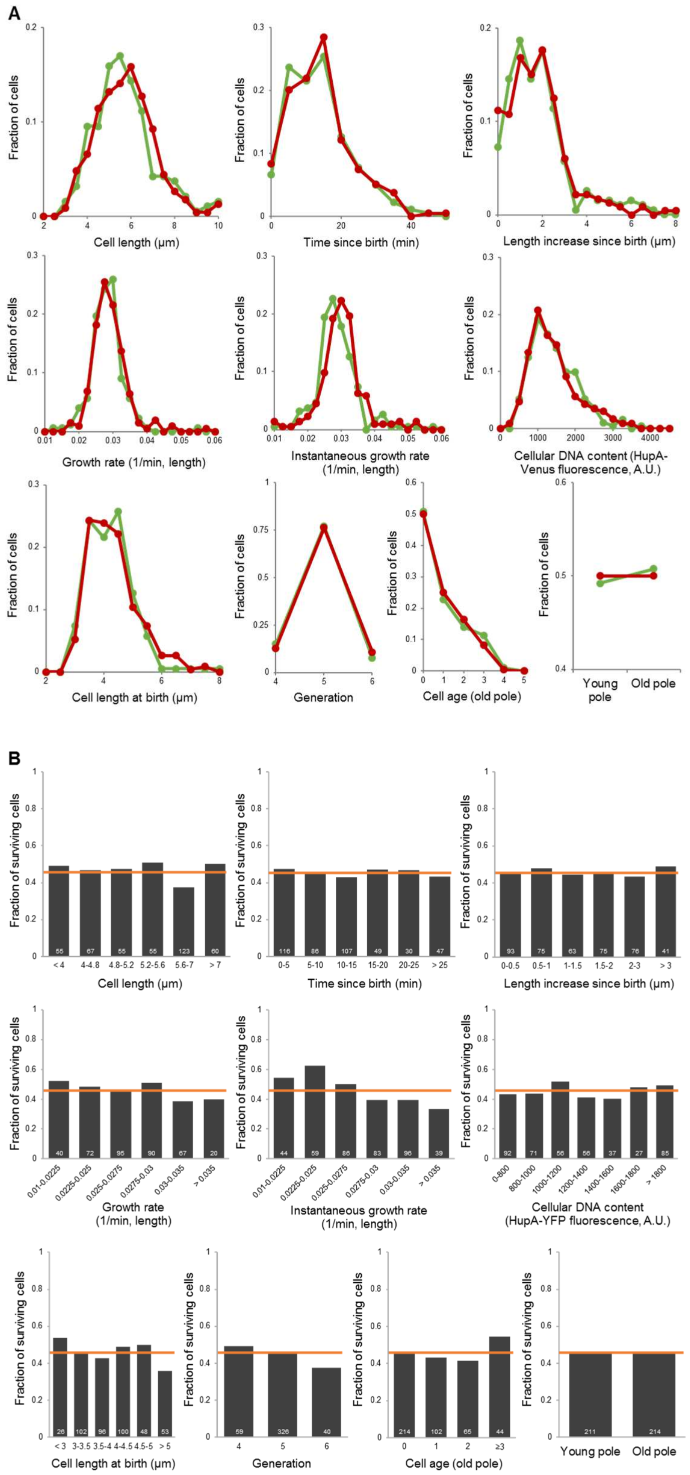
A
Fraction of cells
Cell length (µm)
Time since birth (min)
Length increase since birth (µm)
Growth rate (1/min, length)
Instantaneous growth rate (1/min, length)
Cellular DNA content (HupA-Venus fluorescence, A.U.)
Cell length at birth (µm)
Generation
Cell age (old pole)
Young pole
Old pole
B
Fraction of surviving cells
Cell length (µm)
Time since birth (min)
Length increase since birth (µm)
Growth rate (1/min, length)
Instantaneous growth rate (1/min, length)
Cellular DNA content (HupA-YFP fluorescence, A.U.)
Cell length at birth (µm)
Generation
Cell age (old pole)
Young pole
Old pole

Figure S3

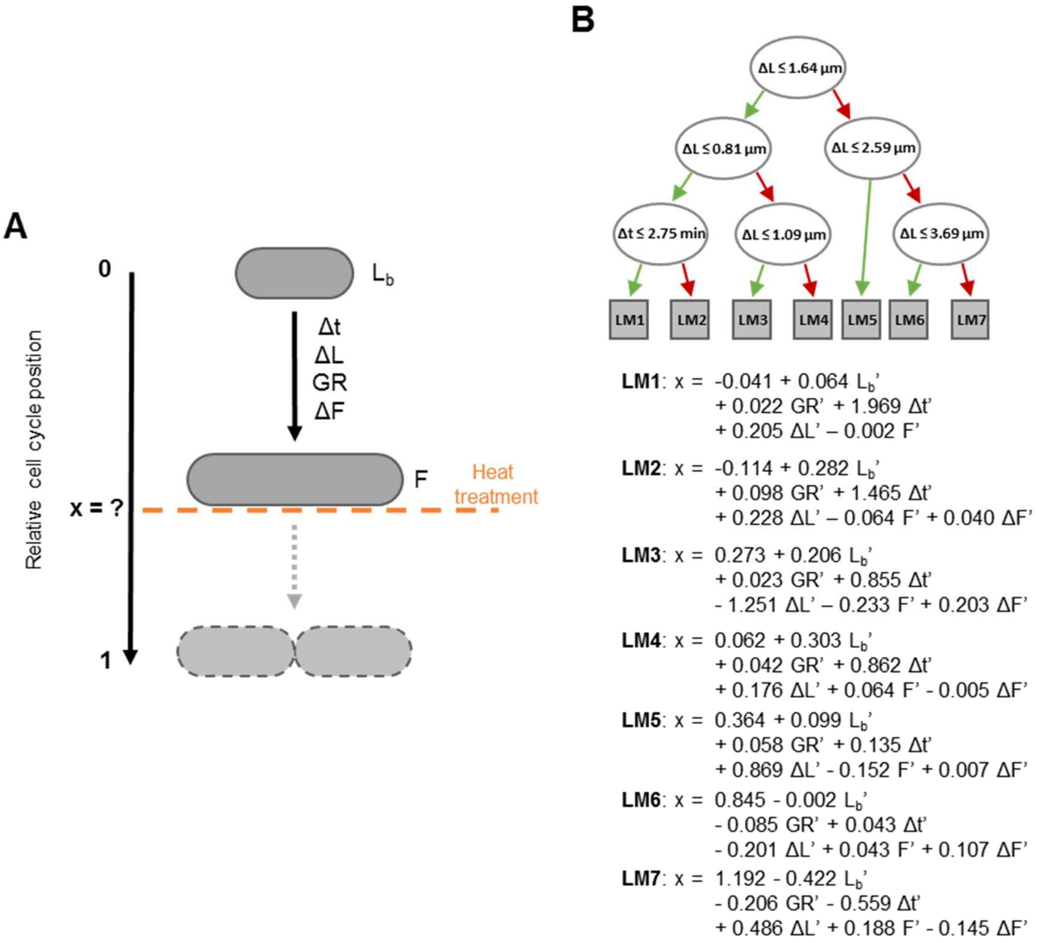

A
0
1
Relative cell cycle position
x = ?
Lb
Δt
ΔL
GR
ΔF
F
Heat treatment
B
ΔL ≤ 1.64 µm
ΔL ≤ 0.81 µm
ΔL ≤ 2.59 µm
Δt ≤ 2.75 min
ΔL ≤ 1.09 µm
ΔL ≤ 3.69 µm
LM1
LM2
LM3
LM4
LM5
LM6
LM7
LM1: x = -0.041 + 0.064 Lb' + 0.022 GR' + 1.969 Δt' + 0.205 ΔL' – 0.002 F'
LM2: x = -0.114 + 0.282 Lb' + 0.098 GR' + 1.465 Δt' + 0.228 ΔL' – 0.064 F' + 0.040 ΔF'
LM3: x = 0.273 + 0.206 Lb' + 0.023 GR' + 0.855 Δt' - 1.251 ΔL' – 0.233 F' + 0.203 ΔF'
LM4: x = 0.062 + 0.303 Lb' + 0.042 GR' + 0.862 Δt' + 0.176 ΔL' + 0.064 F' - 0.005 ΔF'
LM5: x = 0.364 + 0.099 Lb' + 0.058 GR' + 0.135 Δt' + 0.869 ΔL' - 0.152 F' + 0.007 ΔF'
LM6: x = 0.845 - 0.002 Lb' - 0.085 GR' + 0.043 Δt' - 0.201 ΔL' + 0.043 F' + 0.107 ΔF'
LM7: x = 1.192 - 0.422 Lb' - 0.206 GR' - 0.559 Δt' + 0.486 ΔL' + 0.188 F' - 0.145 ΔF'

Figure S4

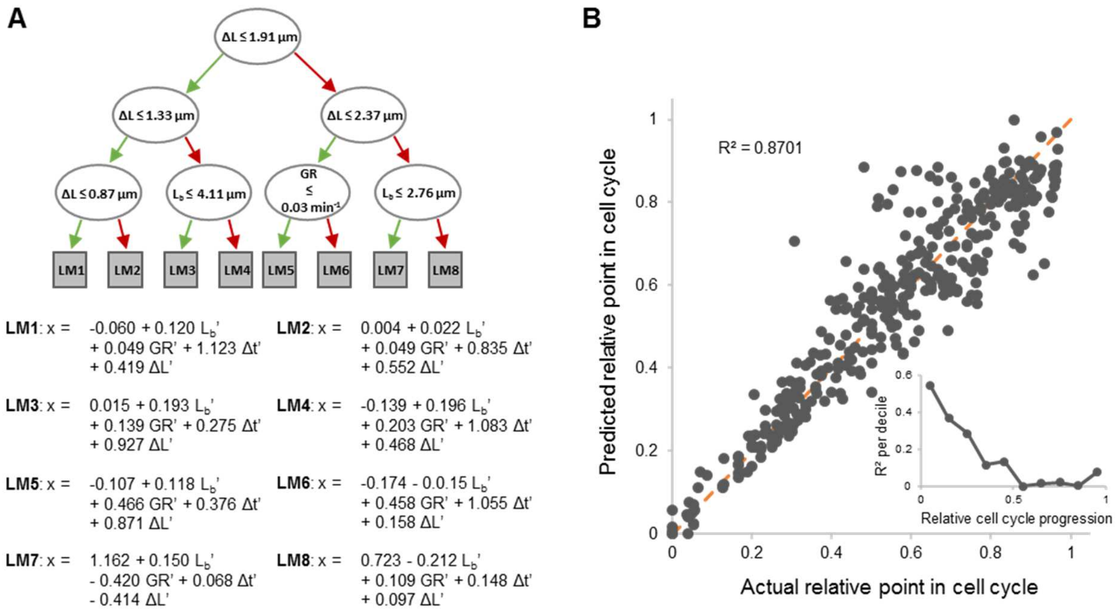
A
ΔL ≤ 1.91 μm
ΔL ≤ 1.33 μm
ΔL ≤ 2.37 μm
ΔL ≤ 0.87 μm
Lb ≤ 4.11 μm
GR ≤ 0.03 min-1
Lb ≤ 2.76 μm
LM1
LM2
LM3
LM4
LM5
LM6
LM7
LM8
LM1: x = -0.060 + 0.120 Lb' + 0.049 GR' + 1.123 Δt' + 0.419 ΔL'
LM2: x = 0.004 + 0.022 Lb' + 0.049 GR' + 0.835 Δt' + 0.552 ΔL'
LM3: x = 0.015 + 0.193 Lb' + 0.139 GR' + 0.275 Δt' + 0.927 ΔL'
LM4: x = -0.139 + 0.196 Lb' + 0.203 GR' + 1.083 Δt' + 0.468 ΔL'
LM5: x = -0.107 + 0.118 Lb' + 0.466 GR' + 0.376 Δt' + 0.871 ΔL'
LM6: x = -0.174 - 0.0.15 Lb' + 0.458 GR' + 1.055 Δt' + 0.158 ΔL'
LM7: x = 1.162 + 0.150 Lb' - 0.420 GR' + 0.068 Δt' - 0.414 ΔL'
LM8: x = 0.723 - 0.212 Lb' + 0.109 GR' + 0.148 Δt' + 0.097 ΔL'
B
R² = 0.8701
Predicted relative point in cell cycle
Actual relative point in cell cycle
R² per decile
Relative cell cycle progression

Figure S5

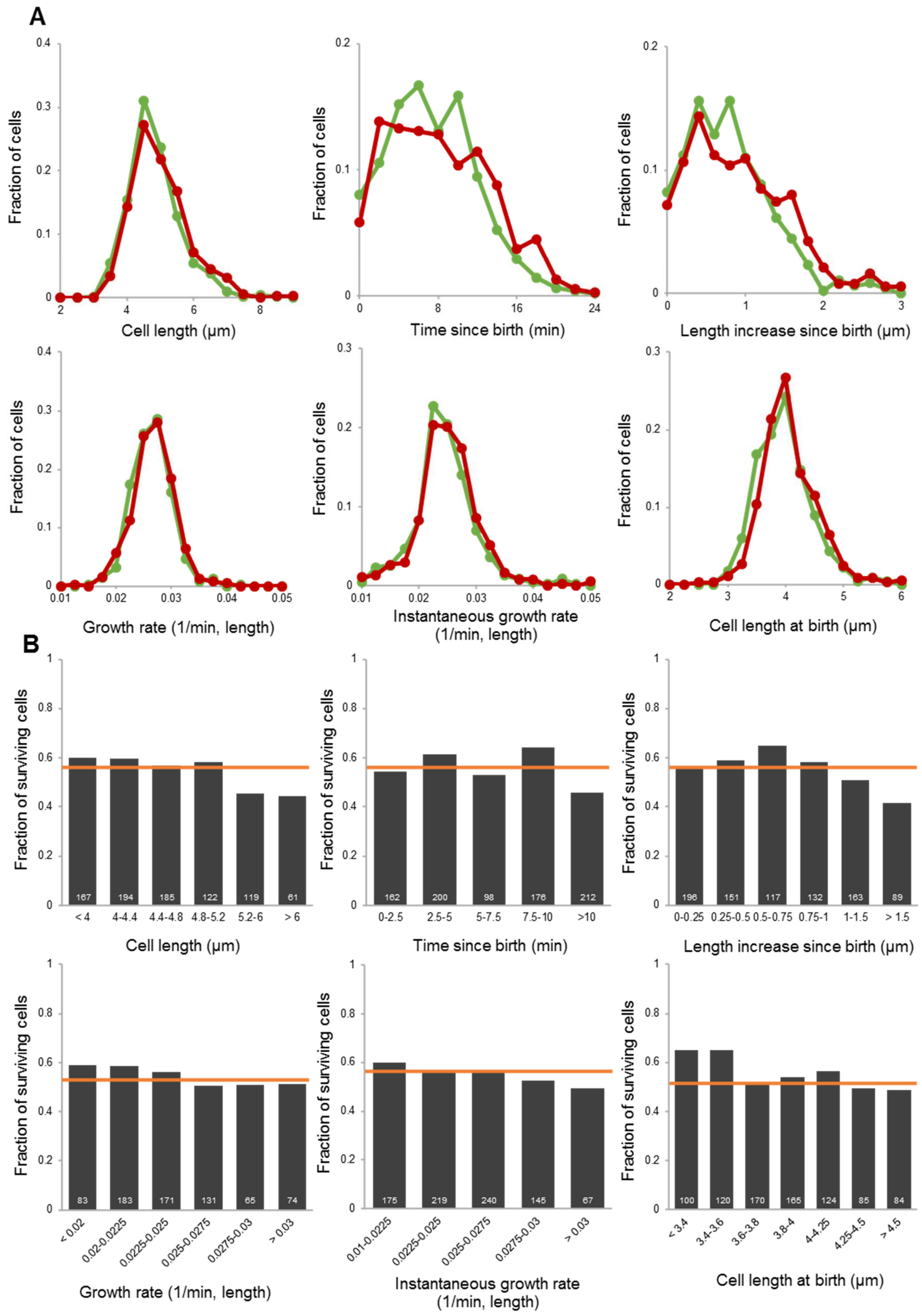
A
Fraction of cells
Cell length (µm)
Time since birth (min)
Length increase since birth (µm)
Growth rate (1/min, length)
Instantaneous growth rate (1/min, length)
Cell length at birth (µm)
B
Fraction of surviving cells
167 194 185 122 119 61
< 4 4-4.4 4.4-4.8 4.8-5.2 5.2-6 > 6
Cell length (µm)
162 200 98 176 212
0-2.5 2.5-5 5-7.5 7.5-10 >10
Time since birth (min)
196 151 117 132 163 89
0-0.25 0.25-0.5 0.5-0.75 0.75-1 1-1.5 > 1.5
Length increase since birth (µm)
83 183 171 131 65 74
< 0.02 0.02-0.0225 0.0225-0.025 0.025-0.0275 0.0275-0.03 > 0.03
Growth rate (1/min, length)
175 219 240 145 67
0.01-0.0225 0.0225-0.025 0.025-0.0275 0.0275-0.03 > 0.03
Instantaneous growth rate (1/min, length)
100 120 170 165 124 85 84
< 3.4 3.4-3.6 3.6-3.8 3.8-4 4-4.25 4.25-4.5 > 4.5
Cell length at birth (µm)

Figure S6

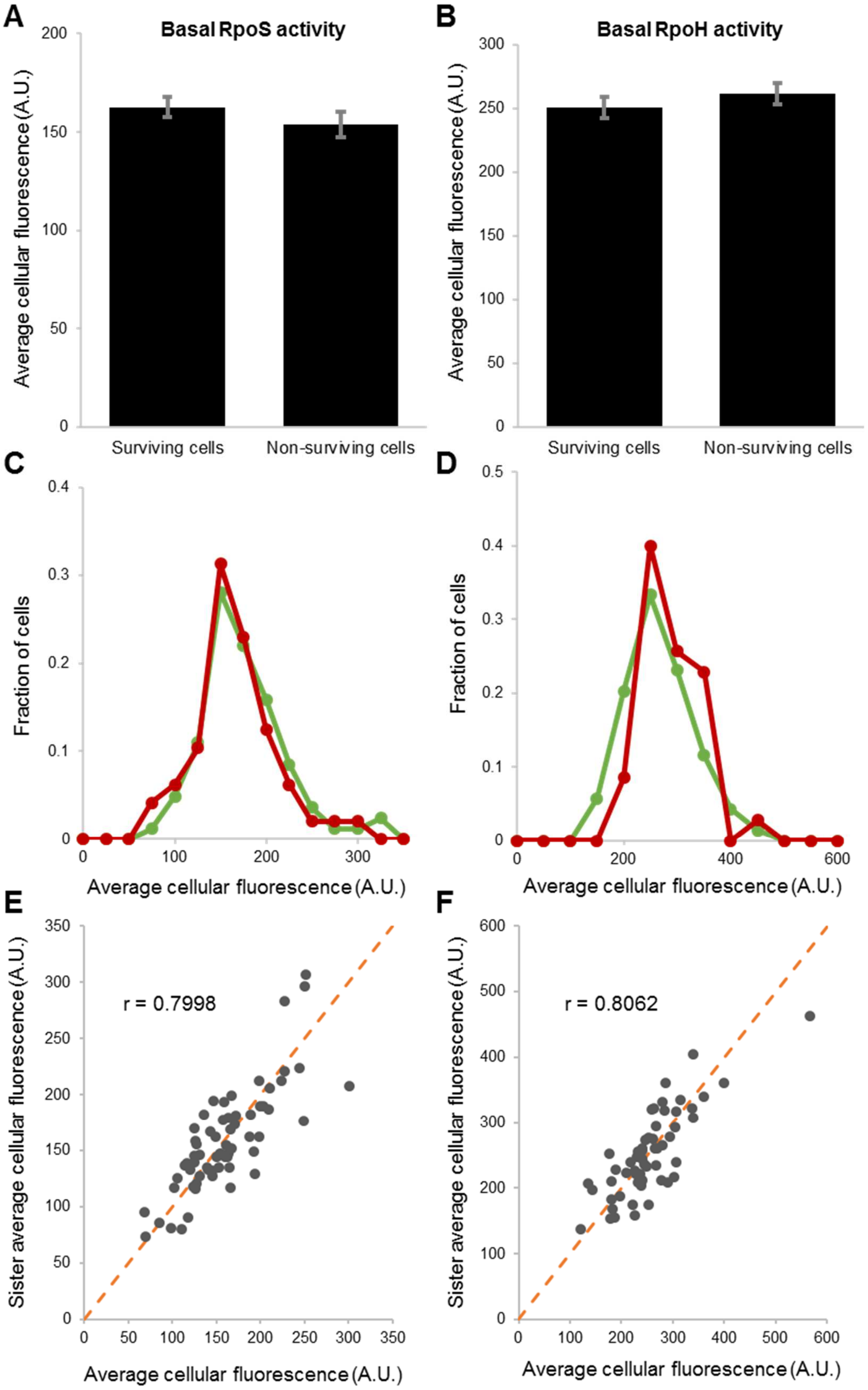
A
Basal RpoS activity
Average cellular fluorescence (A.U.)
Surviving cells
Non-surviving cells
B
Basal RpoH activity
Average cellular fluorescence (A.U.)
Surviving cells
Non-surviving cells
C
Fraction of cells
Average cellular fluorescence (A.U.)
D
Fraction of cells
Average cellular fluorescence (A.U.)
E
r = 0.7998
Sister average cellular fluorescence (A.U.)
Average cellular fluorescence (A.U.)
F
r = 0.8062
Sister average cellular fluorescence (A.U.)
Average cellular fluorescence (A.U.)

Figure S7

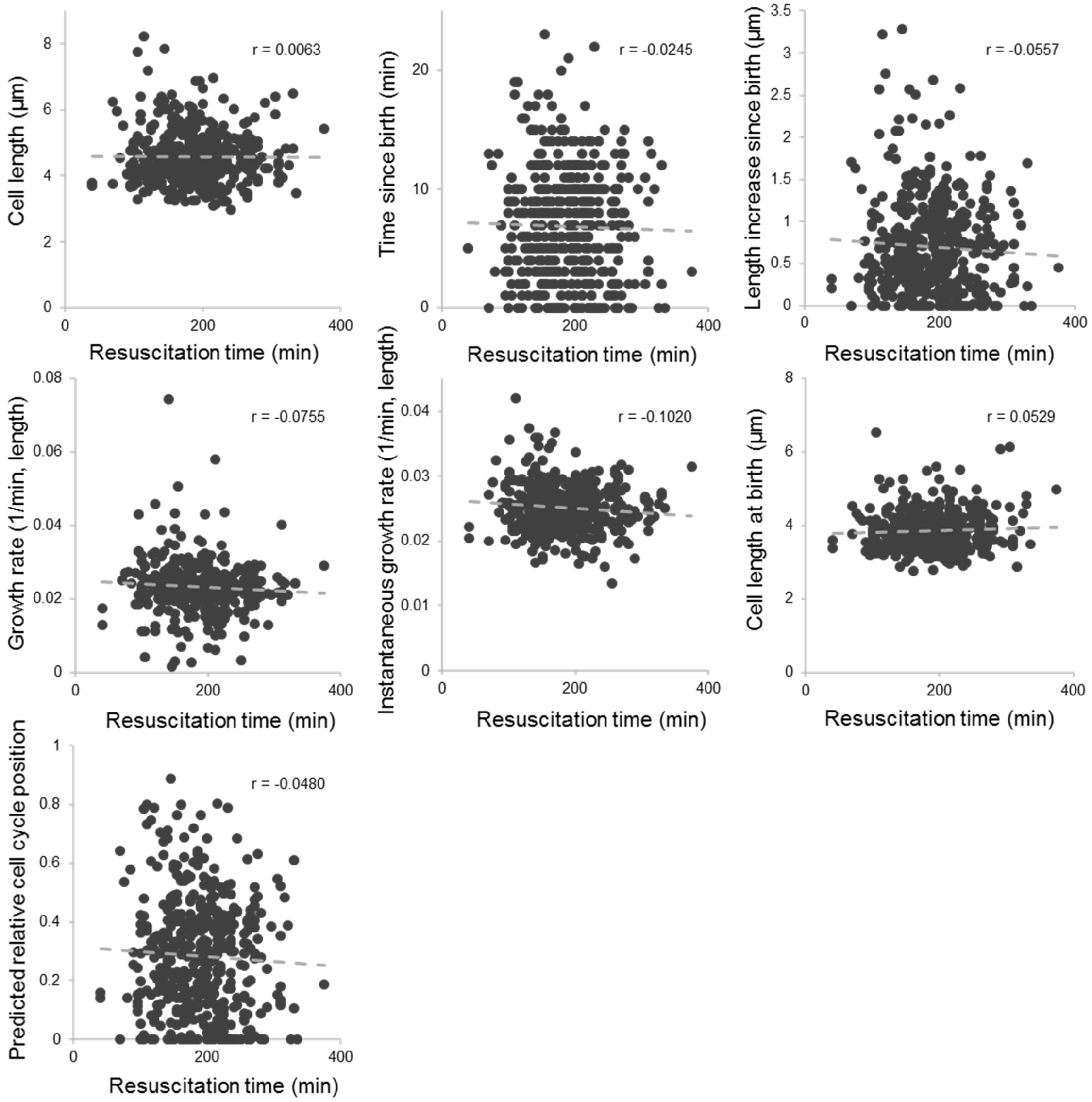

Cell length (µm)
r = 0.0063
Resuscitation time (min)
Time since birth (min)
r = -0.0245
Resuscitation time (min)
Length increase since birth (µm)
r = -0.0557
Resuscitation time (min)
Growth rate (1/min, length)
r = -0.0755
Resuscitation time (min)
Instantaneous growth rate (1/min, length)
r = -0.1020
Resuscitation time (min)
Cell length at birth (µm)
r = 0.0529
Resuscitation time (min)
Predicted relative cell cycle position
r = -0.0480
Resuscitation time (min)

Figure S8

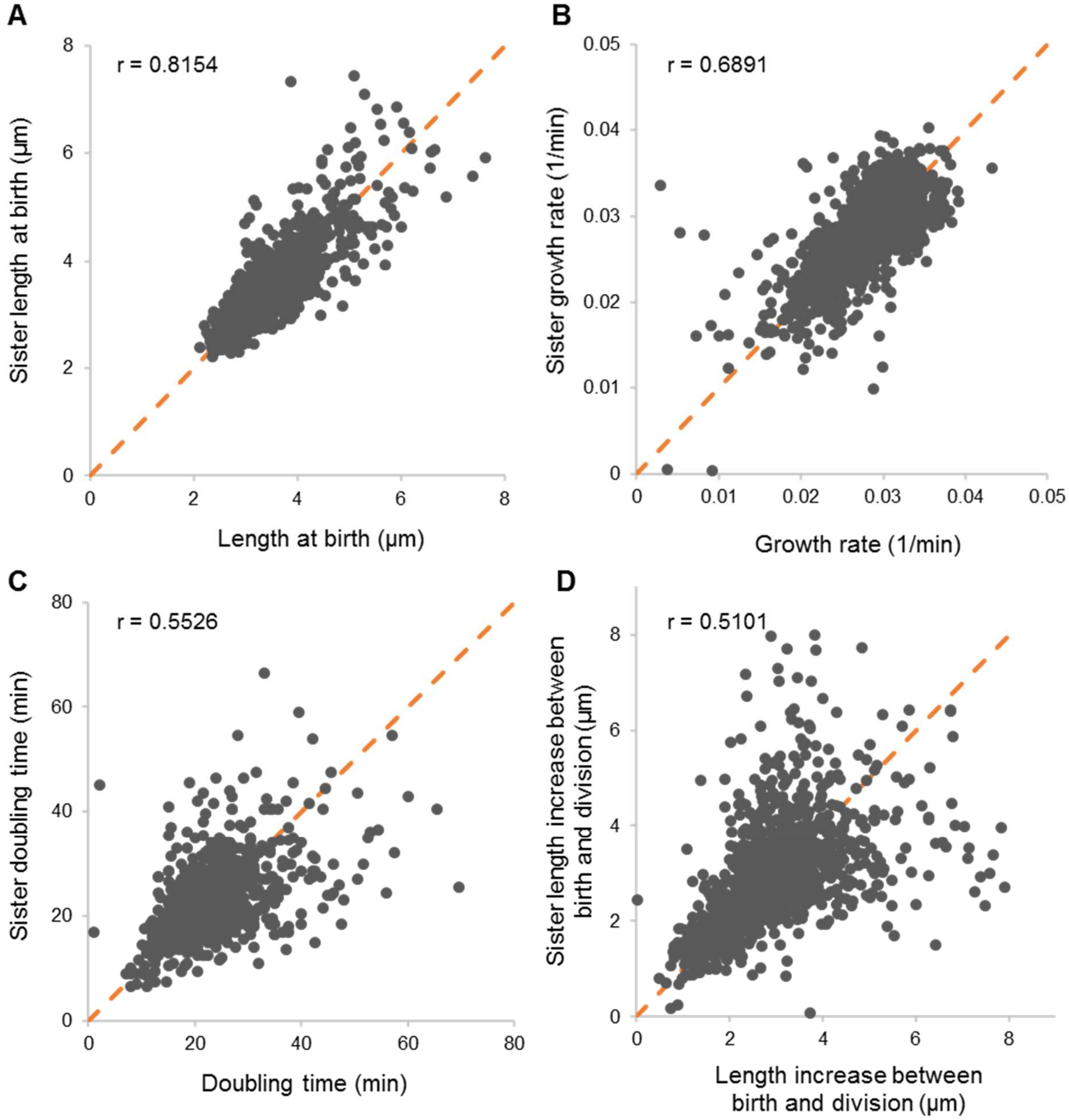
A
r = 0.8154
Sister length at birth (µm)
Length at birth (µm)
B
r = 0.6891
Sister growth rate (1/min)
Growth rate (1/min)
C
r = 0.5526
Sister doubling time (min)
Doubling time (min)
D
r = 0.5101
Sister length increase between birth and division (µm)
Length increase between birth and division (µm)
